# Image Fusion During Standard and Complex Endovascular Aortic Repair, to Fuse or Not to Fuse? A Meta-analysis and Additional Data From a Single-Center Retrospective Cohort

**DOI:** 10.1177/1526602820960444

**Published:** 2020-09-23

**Authors:** Sabrina A. N. Doelare, Stefan P. M. Smorenburg, Theodorus G. van Schaik, Jan D. Blankensteijn, Willem Wisselink, Johanna H. Nederhoed, Rutger J. Lely, Arjan W. J. Hoksbergen, Kak Khee Yeung

**Affiliations:** 1 Department of Surgery, Amsterdam Cardiovascular Sciences, Amsterdam UMC, Vrije Universiteit, Amsterdam, the Netherlands; 2 Department of Physiology, Amsterdam Cardiovascular Sciences, Amsterdam UMC, Vrije Universiteit, Amsterdam, the Netherlands; 3 Department of Radiology, Amsterdam Cardiovascular Sciences, Amsterdam UMC, Vrije Universiteit, Amsterdam, the Netherlands

**Keywords:** contrast volume, endovascular aneurysm repair, fenestrated/branched EVAR, fluoroscopy time, fusion imaging, image fusion, meta-analysis, procedure time, radiation dose, systematic review

## Abstract

**Purpose:**

To determine if image fusion will reduce contrast volume, radiation dose, and fluoroscopy and procedure times in standard and complex (fenestrated/branched) endovascular aneurysm repair (EVAR).

**Materials and Methods:**

A search of the PubMed, Embase, and Cochrane databases was performed in December 2019 to identify articles describing results of standard and complex EVAR procedures using image fusion compared with a control group. Study selection, data extraction, and assessment of the methodological quality of the included publications were performed by 2 reviewers working independently. Primary outcomes of the pooled analysis were contrast volume, fluoroscopy time, radiation dose, and procedure time. Eleven articles were identified comprising 1547 patients. Data on 140 patients satisfying the study inclusion criteria were added from the authors’ center. Mean differences (MDs) are presented with the 95% confidence interval (CI).

**Results:**

For standard EVAR, contrast volume and procedure time showed a significant reduction with an MD of −29 mL (95% CI −40.5 to −18.5, p<0.001) and −11 minutes (95% CI −21.0 to −1.8, p<0.01), respectively. For complex EVAR, significant reductions in favor of image fusion were found for contrast volume (MD −79 mL, 95% CI −105.7 to −52.4, p<0.001), fluoroscopy time (MD −14 minutes, 95% CI −24.2 to −3.5, p<0.001), and procedure time (MD −52 minutes, 95% CI −75.7 to −27.9, p<0.001).

**Conclusion:**

The results of this meta-analysis confirm that image fusion significantly reduces contrast volume, fluoroscopy time, and procedure time in complex EVAR but only contrast volume and procedure time for standard EVAR. Though a reduction was suggested, the radiation dose was not significantly affected by the use of fusion imaging in either standard or complex EVAR.

## Introduction

In the past years, imaging capabilities in the hybrid operating room have been profoundly upgraded. Standard angiography has been complemented with 3-dimensional (3D) patient-specific roadmap functionality. Image fusion merges preoperative imaging such as computed tomography angiography (CTA) with live intraoperative fluoroscopy. The operator navigates the guidewires, catheters, and sheaths guided by a 3D roadmap to enable a more accurate and easier deployment of fenestrated and branched endovascular devices and cannulation of visceral arteries.^1^

Several studies showed that complex (fenestrated/branched) EVAR is associated with a significant risk of acute renal failure due to the large volumes of contrast material used. Although the etiology of this problem is likely multifactorial, contrast-enhanced examinations are still the third leading cause of hospital-acquired acute renal failure.^2,3^ Therefore, any effort supporting a reduction in the volume of contrast media use in complex EVAR is considered highly relevant. Additionally, the cumulative effect of exposure to radiation puts patients and physicians at risk for deterministic and stochastic radiation injuries. Several strategies such as the “as low as reasonably achievable” (ALARA) principle have been proposed to minimize the risks of intraoperative radiation. However, these approaches have proved to be insufficient to reduce all risks and are subject to hospital clinical practice.^4^ Hence, new imaging approaches should be used to further reduce contrast and radiation exposure. Furthermore, longer procedure times involved in complex EVAR expose the patient to longer anesthesia, resulting in a prolonged recovery.^5^

Superior clinical outcomes with image fusion have been described in complex EVAR.^4,6–10^ However, there is no general agreement about the need for image fusion in standard EVAR. To the best of our knowledge only 2 meta-analyses concerning image fusion in EVAR have been conducted.^11,12^ One pooled only the data on administered contrast volume and the other contained limited image fusion cohorts. Nonetheless, no meta-analysis has been performed that pooled the data for all procedure metrics (contrast volume, radiation dose, and fluoroscopy and procedure times) for image fusion in both standard and complex EVAR. Furthermore, newer studies are available that can elucidate the effect of image fusion during standard EVAR.

To investigate the hypothesis that image fusion will reduce relevant imaging-related parameters in complex EVAR but not in standard EVAR, a systematic review and meta-analysis was conducted comparing complex to standard EVAR in terms of the aforementioned procedure parameters. To augment the limited studies available describing image fusion in standard EVAR, a cohort of EVAR patients from our hospital was included.

## Materials and Methods

### Study Design

A literature review was performed according to the Preferred Reporting Items for Systematic Reviews and Meta-Analyses (PRISMA) guidelines^13^ to identify clinical studies describing results after image fusion during standard and complex EVAR. The search of the PubMed, Embase, and Cochrane databases was performed by a clinical librarian in December 2019. A broad search was created with the following MeSH terms: “endovascular procedures,” “fusion,” and “imaging”; the full electronic search strategy can be found in Appendix A. Only publications in the English language were selected.

Studies involving traumatic cerebral aneurysms, pediatric patients, aortic dissections, or the use of ultrasound imaging or open surgery were excluded, as were studies without preoperative CT or magnetic resonance (MR) imaging confirmation of the abdominal aortic aneurysm. Studies with <10 patients and reviews, letters, and conference abstracts were excluded. Two reviewers (S.D. and T.v.S.) performed eligibility assessments independently. Disagreements between reviewers were resolved by consensus.

Main outcome measures were the amount of iodinated contrast administered (mL), fluoroscopy time (minutes), cumulative radiation dose expressed in dose area product (DAP, Gy·cm^2^) or air kerma (AK, mGy), and procedure time (minutes). The secondary outcome was clinical success, which was defined as procedure success, aneurysm shrinkage, and no postoperative mortality.

The risk of bias was assessed for each study using the Methodological Index for Non-Randomized Studies (MINORS) scoring system.^14^ Two reviewers (S.D. and T.v.S) independently scored the articles; disagreements were resolved by consensus or consultation with a third reviewer. The MINORS system involves 12 items with a maximum global score of 24 for comparative studies. Each item was scored from 0 to 2. The items were scored 0 if not reported, 1 when reported but inadequate, and 2 when reported and adequate.

### Additional Hospital Data

To supplement image fusion data for the pooled analysis, clinical data were retrieved on 61 consecutive patients treated using standard EVAR with (n=20) or without (n=41) fusion imaging at the discretion of the surgeon between March 2017 and March 2019. Another 79 patients undergoing complex EVAR (37 fusion vs 42 no fusion) between September 2010 and March 2019 were also included. Patient baseline characteristics are stated in Table 1. Details of preoperative imaging acquisition and postprocessing, 2D-3D (standard EVAR) and 3D-3D (complex) registration, and fusion imaging are provided in Appendix B.

**Table 1.** Baseline Characteristics of the Hospital Cohort.^a^

	Standard EVAR (n=61)			Complex EVAR (n=79)		
	Fusion (n=20)	No Fusion (n=41)	p^b^	Fusion (n=37)	No Fusion (n=42)	p^b^
Men	20 (100)	39 (95)	0.04	30 (81)	33 (79)	0.584
Age, y	72.9±6.1	73.1±7.5	0.228	73.2±6.5	72.7±6.6	0.994
BMI, kg/m^2^	28.8±5.3	27.72±5.0	0.449	26.6±4.4	26.4±4.8	0.779

Abbreviations: BMI, body mass index; EVAR, endovascular aneurysm repair.

a Continuous data are presented as the means ± standard deviation; categorical data are given as the counts (percentage).

b Levene test for equality of variances.

### Statistical Analysis

Data on each outcome measure were pooled to generate standardized mean differences (MDs), which were compared using the *t* test for equality of means. For studies that reported only medians with interquartile range, the mean ± standard deviation was calculated using the methods proposed by Wan et al.^15^ In meta-analysis, the studies were compared for contrast use, fluoroscopy time, radiation dose, and procedure time using the inverse variance method in random-effects models. Heterogeneity was assessed using forest plot analysis with the *I*^2^ index.^16^ The meta-analysis was performed in Review Manager (version 5.3.5; The Cochrane Collaboration, Copenhagen, Denmark).

## Results

### Characteristics of the Included Studies

**Figure 1.**
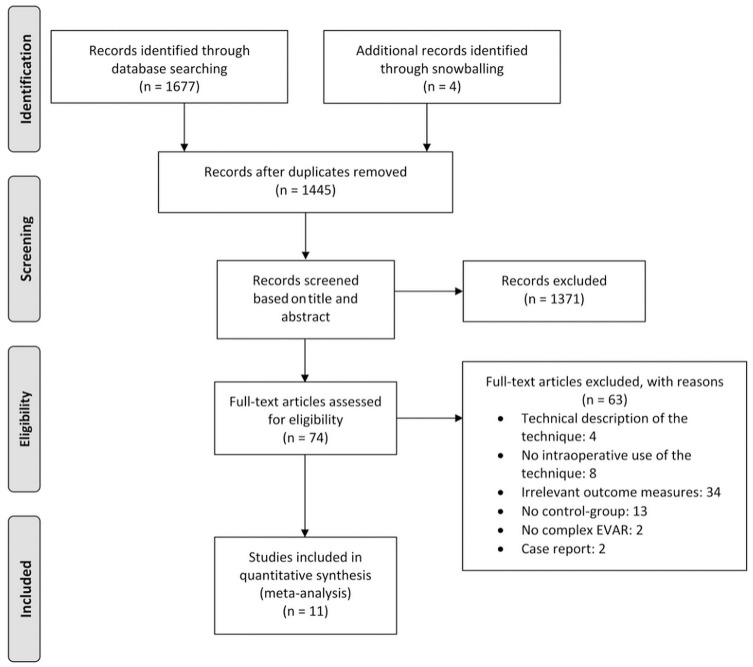
Flowchart of the search strategy. EVAR, endovascular aneurysm repair.

Of the 11 studies selected for analysis (Figure 1), 7 were retrospective cohort studies^6–8,10,17,18,21^ and 4 were prospective cohort studies.^4,9,19,20^ Preoperative CTA was performed in all studies for fusion guidance except for Stangenberg et al,^21^ who performed preoperative MR angiography in one of their 23 patients. A variety of fusion software packages were employed: Xtra Vision^9,10^ (Philips, Best, the Netherlands), Syngo X-Workplace^6–8^ (Siemens Healthineers, Erlangen, Germany), VesselNavigator^17,21^ (Philips), RTRS EV^20^ (Cydar Medical, Barrington, UK), EndoNaut^19^ (Therenva, Rennes, France), Innova Vision/Heart^4^ (GE Healthcare, Chalfont St Giles, UK), and Infinix Vc-I^18^ (Toshiba, Medical Systems, Tokyo, Japan). The study descriptions of the included articles are summarized in Table 2.

**Table 2.** Characteristics and Outcomes of the Included Studies.

First Author, Year	N	Study Design	Preoperative Imaging	Fusion Type (Vendor)	Procedure (Fusion vs No Fusion)	Contrast Volume, mL^a^	Fluoroscopy Time, min^a^	Radiation Dose, DAP (Gy⋅cm^2^) or AK (mGy)^a^	Procedure Time, min^a^	Clinical Success, %
Ahmad, 2018^17^	152	R	CTA	2FP 2D-3D (Philips)	EVAR (n=105 vs n=47)	50 (38–59) vs 72 (60–102), p<0.001	6.3 (5.0–9.0) vs 9.5 (6.2–12.5), p=0.067	DAP: 234.4 (158–514) vs 321.9 (143.1–494.2), p=0.457	64.5 (59–68) vs 83 (68–89), p=0.002	—
Dias, 2015^6^	226	R	CTA	CBCT 3D-3D (Siemens)	TEVAR (n=10 vs n=15)	53 (50–175) vs 178 (128–282), p=0.4	14 (9–31) vs 28 (19–39), p=0.688	DAP: 90.4 (61.9–158.5) vs 173.0 (143.7–386.3), p=0.041	83 (56–183) vs 144 (95–144), p=0.459	80 vs 100, p=0.150
					f/bEVAR (n=33 vs n=23)	143 (196–197) vs 298 (234–363), p<0.001	103 (84–139) vs 127 (104–157), p=0.103	DAP: 262.9 (203.0–367.7) vs 638.9 (436.9–1002.7), p<0.001	349 (261–438) vs 459 (391–607), p=0.007	94% vs 87%, p=0.634
					fEVAR (n=21 vs n=36)	96 (69–144) vs 208 (162–238), p<0.001	72 (48–131) vs 87 (58–99), p=0.581	DAP: 241.7 (140.4–432.0) vs 283.2 (192.1–499.6), p=0.581	231 (163–332) vs 277 (193–374), p=0.319	95 vs 92, p>0.99
					EVAR (n=22 vs n=25)	84 (49–136) vs 160 (146–204), p<0.001	25 (18–40) vs 38 (30–45.3), p=0.185	DAP: 98.9 (83.6–164.7) vs 213.8 (124.0–290.1), p=0.013	128 (102–151) vs 149 (119–193), p=0.459	96 vs 96, p>0.99
					IBD (n=17 vs n=24)	132 (85–185) vs 255 (224–312), p<0.001	57 (35–76) vs 80 (60–101), p=0.077	DAP: 188.7 (121.9–234.9) vs 468.7 (328.9–617.1), p<0.001	191 (130–258) vs 249 (191–281), p=0.131	88 vs 88, p>0.99
					Combined (n=103 vs n=123)	103 (63–145) vs 215 (166–280), p<0.001	66 (33–102) vs 72 (42–102), p=0.837	DAP: 199.3 (113.4–306.2) vs 328.6 (195.6–5568.8), p<0.001	230 (134–331) vs 235 (158–364), p=0.942	92 vs 92, p>0.99
Dijkstra, 2011^7^	89	R	CT	CBCT 3D-3D (Siemens)	fEVAR (n=40 vs n=49)	94 (72–131) vs 136 (96–199), p=0.001	81 (54–118) vs 90 (46–128), p=0.932	AK: 7 (4–12) vs 7 (5–10), p=0.782	330 (273–522) vs 387 (290–477), p=0.651	85 vs 90, p=0.975
Hertault, 2014^4^	397	P	CTA	2FP 2D-3D (GE Healthcare)	EVAR (n=44 vs n=199)	59 (50–75) vs 80 (65–106), p=0.34	—	DAP: 12.2 (8.7–19.9) vs 30.0 (20.0–43.5), p<0.01	92.5 (75–120) vs 93 (75–120), p=0.97	—
					bEVAR (n=20 vs n=20)	120 (100–170) vs 138 (100–160), p<0.01	—	DAP: 47.4 (37.2–108.2) vs 159.0 (101.8–222.4), p<0.01	205(169–240) vs 210 (150–260), p=0.87	—
					fEVAR (n=18 vs n=54)	105 (70–136) vs 138 (100–160), p=0.03	—	DAP: 43.7 (24.7–57.5) vs 72.9 (52–109.2), p<0.01	150 (150–160) vs 150 (105–180), p=0.39	—
					TEVAR (n=14 vs n=28)	80 (50–100) vs 100 (78–140), p=0.07	—	DAP: 24.7 (22.0–28.7) vs 20.0 (11.4–30.0), p=0.63	80 (60–105) vs 117 (60–138), p=0.22	—
Hiraoka, 2018^18^	143	R	CTA	2FP 2D-3D (Toshiba)	EVAR (n=81 vs n=62)	76.2±27.6 vs 89.0±28.5, p=0.009	—	AK: 768±529 vs 880±833, p=0.333	116±27 vs 113±37, p=0.487	—
					TEVAR (n=83 vs n=37)	72.6±21.1 vs 85.6±31.1, p=0.009	—	AK: 638±463 vs 872±623, p=0.033	86.2±23.9 vs 96.4±27, p=0.023	—
Kaladji, 2018^19^	152	P	CTA	2FP 2D-3D (Therenva)	EVAR (n=49 vs n=103)	42.3±22.0 vs 81.2±48.0 p<0.001	21.9±12.0 vs 19.5±13.0 p=0.27	DAP: 70.6±4.8 vs 67.3±74, p=0.77	114±44 vs 140.8±38, p<0.001	—
Maurel, 2018^20^	65	P	CTA	2FP 2D-3D (Cydar)	EVAR (n=44 vs n=21)	45 (36–60) (no control group)	29.5 (22–33) vs 32 (23–38), p=0.36	AK: 82 (51–115) vs 142 (61–541), p=0.028 DAP: 12.37 (7.48–23.63) vs 21.73 (8.92–85.94), p=0.11	90 (75–100) vs 90 (75–110), p=0.56	91 vs 90.5, no p-value
McNally, 2015^8^	72	R	CTA	CBCT 3D-3D (Siemens)	fEVAR (n=12 vs n=8)	26±8 vs 69±16, p<0.001	41±11 vs 63±29, p=0.02	DAP: 1380±520 vs 3400±1900, p=0.001	—	—
					fEVAR (n=19 vs n=33)	39±17 vs 90±25, p<0.001	6±21 vs 89±36, p=0.02	DAP: 2700±1400 vs 5400±2225, p<0.001	230±50 vs 330±100, p=0.002	
					Combined (n=31 vs n=41)	34±15 vs 86±25, p<0.001	55±21 vs 84±36, p<0.001	DAP: 2200±1300 vs 5000±280, p<0.001	—	76 vs 84, no p-value
Sailer, 2014^9^	62	P	Dual energy CTA	CBCT 3D-3D (Philips)	f/bEVAR (n=31 vs n=31)	159±71 vs 199±78, p=0.037	50.43± 29.58 vs 59.48± 28.00, p=0.38	—	312±108 vs 378±144, p=0.022	—
Stangenberg, 2015^21^	32	R	CTA, MRA	CBCT 3D-3D/2FP 2D-3D (Philips)	EVAR (n=16 vs n=16)	37.4±21.3 vs 77.3±23.0, p<0.001	18.4±6.8 vs 26.8±10.0, p=0.01	AK: 1067±470.4 vs 1768±696.2, p=0.004	80.4±21.2 vs 110.0±29.1, p=0.005	—
Tacher, 2013^10^	37	R	CTA	CBCT 3D-3D (Philips)	f/bEVAR/ chEVAR (2D: n=9 3D: n=14 IF: n=14)					—
					2D vs 3D vs IF	235±145 vs 225±119 vs 65±28, p<0.001	82±46 vs 42±22 vs 80±36, p=0.04	DAP: 1188±1067 vs 984±581 vs 656±457, p=0.18	223±123 vs 181±53 vs 189±60, p=0.59	89 vs 100 vs 100, p=0.24
					2D vs IF	235±145 vs 65±28, p<0.001	—	DAP: 1188±1067 vs 656±457, p=0.35	—	—
					3D vs IF	225±119 vs 65±28, p<0.001	—	DAP: 984±581 vs 656±457, p=0.06	—	—
Hospital cohort, 2019	40	R	CTA	2FP 2D-3D (Philips)	EVAR (n=20 vs n=41)	105.6±36.2 vs 111.5±51.5, p=0.66	28.8±11.2 vs 29.0±15.9, p=0.79	DAP: 159.1±102.4 vs 139.8±186.8, p=0.67 AK: 810.7±496.7 vs 694.0±913.8, p=0.60	145.0±44.1 vs 138.8±46.8, p=0.62	—
				CBCT 3D-3D	f/bEVAR (n=37 vs n=42)	161.6±59.3 (no control group)	88.1±45.86 (no control group)	DAP: 391.5±348.4 AK: 2337.2±1744.9 (no control group)	268.6±93.1 vs 321.6±154.4, p=0.11	—

Abbreviations: 2D-3D, 2-/3-dimensional angiography; 2FP, 2 fluoroscopic projections; AK, air kerma; bEVAR, branched endovascular aneurysm repair; CBCT, cone-beam computed tomography; chEVAR, chimney endovascular aneurysm repair; CT, computed tomography; CTA, computed tomography angiography; DAP, dose area product; EVAR, endovascular aneurysm repair; f/bEVAR, fenestrated and/or branched EVAR; fEVAR, fenestrated EVAR; IBD, iliac branch device; IF, image fusion; P, prospective; R, retrospective; TEVAR, thoracic endovascular aortic repair.

a Data are presented as the means ± standard deviation or median (interquartile range).

For analysis, the results of Hertault et al,^4^ Dias et al,^6^ and Tacher et al^10^ were subdivided into fenestrated and branched EVAR subgroups. All other complex EVARs were grouped into one cohort containing both fenestrated and branched EVAR cases.

### Risk of Bias

Table 3 shows the assessment of study quality using the MINORS system.^14^ The global scores ranged from 10 to 17 out of 24, indicating a moderate to high degree of bias. All studies lost points for prospective data collection, unbiased assessment of the study endpoints, follow-up period appropriate to the aim of the study, loss to follow-up <5%, prospective power calculation, contemporary groups, and baseline equivalence of groups. Most studies included consecutive patients, adequate control groups, and suitable endpoints, which strengthened confidence in the conclusions.

**Table 3.** MINORS Score.^a^

	Ahmad, 2018^17^	Dias, 2015^6^	Dijkstra, 2011^7^	Hertault, 2014^4^	Hiraoka, 2018^18^	Kaladji, 2018^19^	Maurel, 2018^20^	McNally, 2015^8^	Sailer, 2014^9^	Stangenberg, 2015^21^	Tacher, 2013^10^
A clearly stated aim	2	2	2	2	2	2	2	2	2	2	2
Inclusion of consecutive patients	2	2	2	2	2	2	2	2	2	2	2
Prospective collection of data	0	1	1	2	1	2	2	1	1	1	1
Endpoints appropriate to the aim of the study	1	1	1	2	2	2	2	2	2	2	2
Unbiased assessment of the study endpoint	0	0	0	0	0	0	0	0	0	0	0
Follow-up period appropriate to the aim to the study	0	2	1	0	1	0	2	1	0	0	2
Loss to follow-up <5%	0	0	0	0	1	0	2	0	0	0	0
Prospective calculation of the study size	0	0	0	0	0	0	0	0	0	0	0
An adequate control group	2	2	2	1	2	2	0	1	2	2	2
Contemporary groups	1	1	1	1	2	1	1	1	1	1	1
Baseline equivalence of groups	0	2	2	0	2	2	2	0	0	0	1
Adequate statistical analyses	2	2	2	2	2	2	2	2	2	2	2
Total	10	15	14	13	17	15	17	12	12	12	15

Abbreviation: MINORS, Methodological Index for Non-Randomized Studies.

a MINORS criteria: 0, not reported; 1: reported but inadequate; 2, reported and adequate.^14^

### Contrast Volume

**Figure 2.**
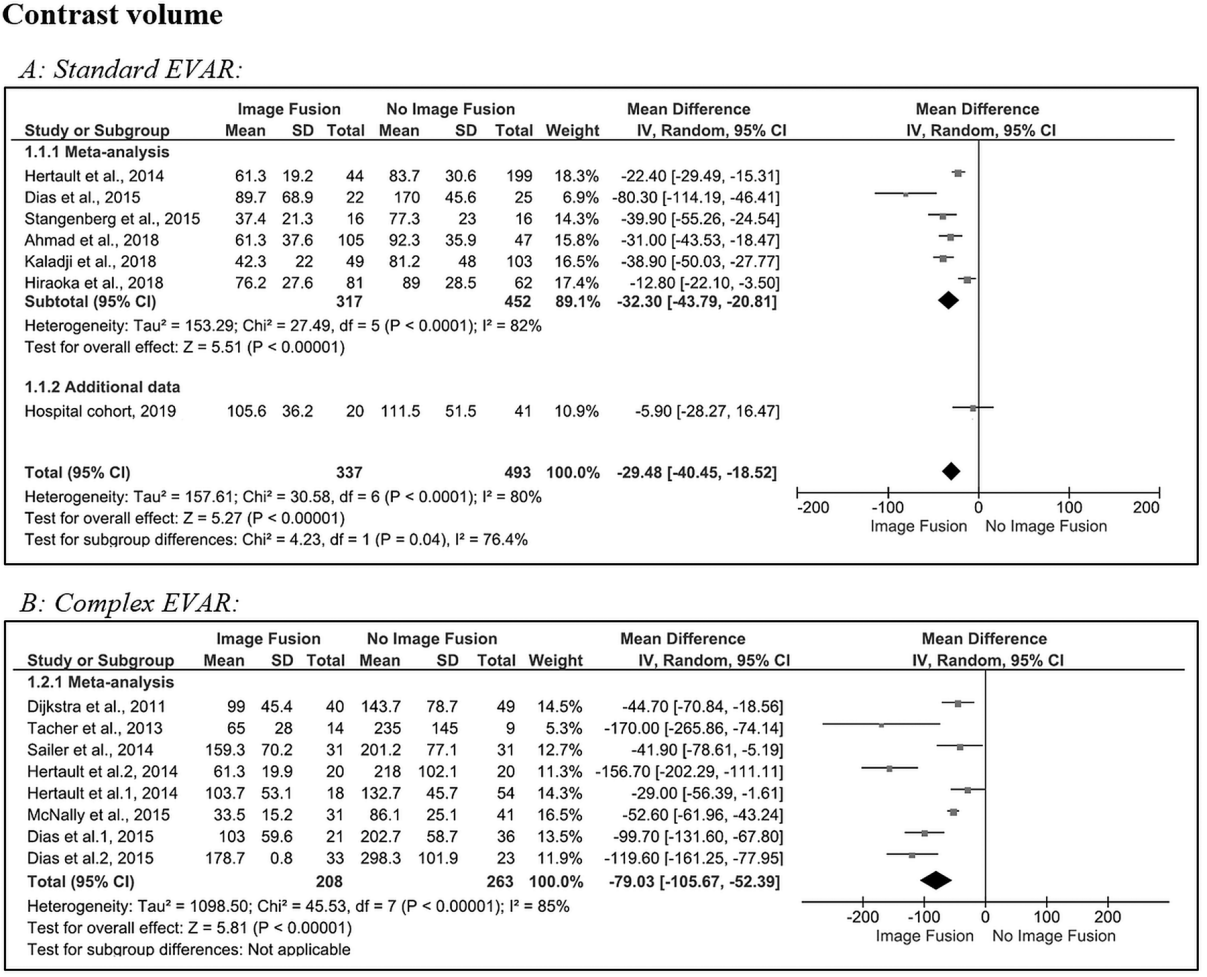
Forest plots of contrast volume for (A) standard and (B) complex endovascular aneurysm repair (subgroups were analyzed for Dias et al^6^ and Hertault et al^4^). CI, confidence interval; IV, inverse variance; SD, standard deviation.

For all endovascular procedures, the contrast volume was reduced in the image fusion group compared to the control group (11 studies).^4,6–10,17–21^ The amount of contrast volume in the control group is not mentioned by Maurel et al.^20^ Figure 2A shows the pooled results for the contrast volume used in standard EVAR procedures. Although heterogeneous (Q=30.6, p<0.001; *I*^2^=80%), the forest plot shows an estimated pooled MD with a significant difference in contrast volume of −29 mL (95% CI −40.5 to −18.5, p<0.001) after image fusion compared with no image fusion.^4,6,17–19,21^ The results of the hospital cohort (–6 mL, 95% CI −28.3 to 16.5, p=0.67) are comparable to Hiraoka et al,^18^ which had the lowest MD among the meta-analyzed studies (–13 mL, 95% CI −22.1 to −3.50, p=0.009) in standard EVAR.

In complex EVAR (Figure 2B), statistical heterogeneity was significant, and the between-study variability was considered high (Q=45.5, p<0.001; *I*^2^=85%) in the 6 included studies.^4,6–10^ The estimated pooled MD (–79 mL, 95% CI −105.7 to −52.4) revealed a significant (p<0.001) reduction in contrast volume after image fusion.

### Fluoroscopy Time

Overall, a statistically significant difference was found in fluoroscopy time between the image fusion group and control group for complex EVAR (5 studies)^6–10^; however, there was no significant difference in standard EVAR (5 studies).^6,17,19–21^ In addition, Stangenberg et al^21^ was the only study reporting a significant fluoroscopy time reduction during standard EVAR, whereas the other 4 studies reported no difference or even an increase in fluoroscopy time as can be deduced from Table 2.

**Figure 3.**
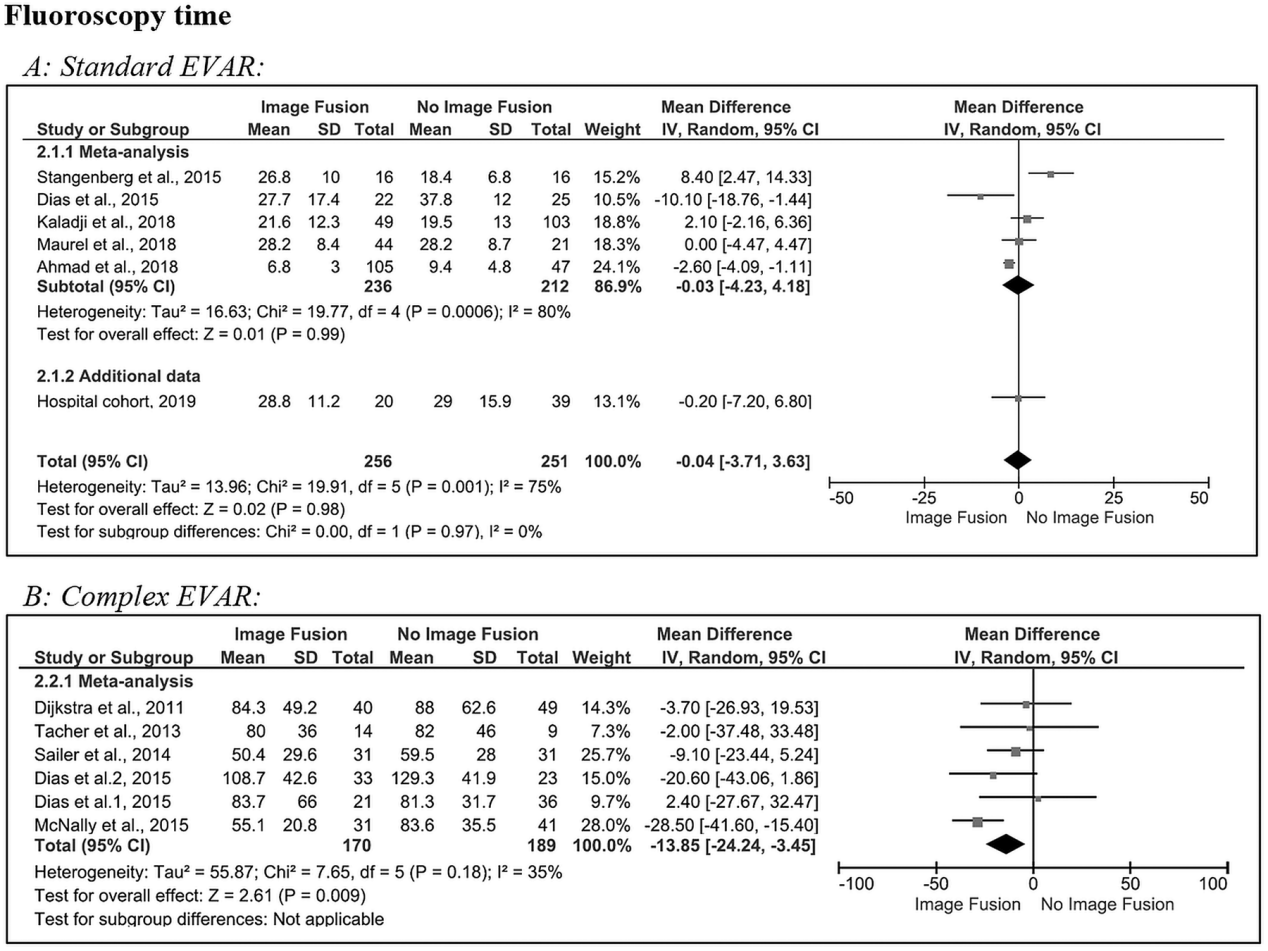
Forest plots of fluoroscopy time for (A) standard and (B) complex endovascular aneurysm repair (subgroups were analyzed for Dias et al^6^). CI, confidence interval; IV, inverse variance; SD, standard deviation.

Figure 3A shows the pooled results for the fluoroscopy time in standard EVAR procedures, which was not different after image fusion compared to no image fusion (0 minutes, 95% CI −3.7 to 3.6, p=0.98). There was significant heterogeneity (Q=19.9, p=0.001; *I*^2^=75%) in the 5 included studies^6,17,19–21^ and the hospital cohort. The MD of the hospital cohort (0 minutes, 95% CI −7.2 to 6.8, p=0.79) was comparable with the meta-analysis subtotal (0 minutes, 95 CI −4.2 to 4.2, p<0.001) during standard EVAR.

In complex EVAR (Figure 3B) statistical heterogeneity was non-significant, and the between-study variability was considered low (Q=7.65, p=0.18; *I*^2^=35%) in the 5 included studies.^6–10^ The estimated pooled MD (–14 minutes, 95% CI −24.2 to −3.5) revealed a significant (p<0.001) reduction in fluoroscopy time after image fusion compared with no image fusion.

### Radiation Dose

**Figure 4.**
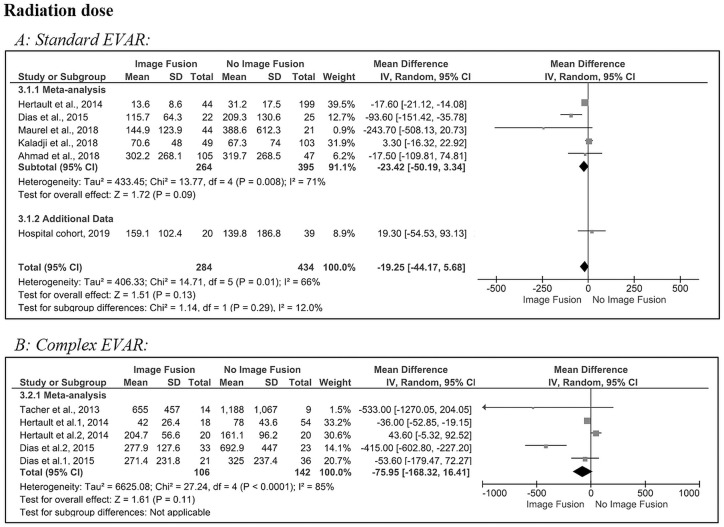
Forest plots of radiation dose (dose area product [DAP], Gy·cm^2^) for (A) standard and (B) complex endovascular aneurysm repair (subgroups were analyzed for Dias et al^6^ and Hertault et al^4^). CI, confidence interval; IV, inverse variance; SD, standard deviation.

The radiation dose (expressed as DAP or AK) was reduced in the image fusion group compared with the control group (6 studies).^4,6,10,17,19,20^ The radiation dose was not described by Sailer et al.^9^ Figure 4A shows the pooled results for the radiation dose used in standard EVAR. The estimated pooled MD revealed a nonsignificant reduction in radiation dose (–19 Gy∙cm^2^, 95% CI −44.2 to 5.7, p=0.13) after image fusion compared to no image fusion. There was a significant heterogeneity (Q=15, p=0.01; *I*^2^=66%) in the 5 included studies^4,6,17,19,20^ and the hospital cohort. The latter had an MD (19 Gy∙cm^2^, 95% CI −54.5 to 93.1, p=0.67) similar to Kaladji et al^19^ (3 Gy∙cm^2^, 95% CI −16.3 to 22.9, p=0.77) for standard EVAR.

In complex EVAR (Figure 4B), there was no significant reduction in radiation dose after image fusion compared with no image fusion (–76 Gy∙cm^2^, 95% CI −168.3 to 16.4, p=0.11). There was significant heterogeneity (Q=27.2, p<0.001; *I*^2^=85%) in the 3 included studies.^4,6,10^

### Procedure Time

There was a statistically significant difference in procedure time between the image fusion group and control group (11 studies).^4,6–10,17–21^ As can be seen in Table 2, a statistically significant difference in procedure time between the image fusion and no fusion groups was found in 7 studies.^6,8,9,17–19,21^ In 2 studies, the procedure times were significantly reduced in only a subgroup (the f/bEVAR subgroup described by Dias et al^6^ and the TEVAR subgroup of Hiraoka et al^18^).

**Figure 5.**
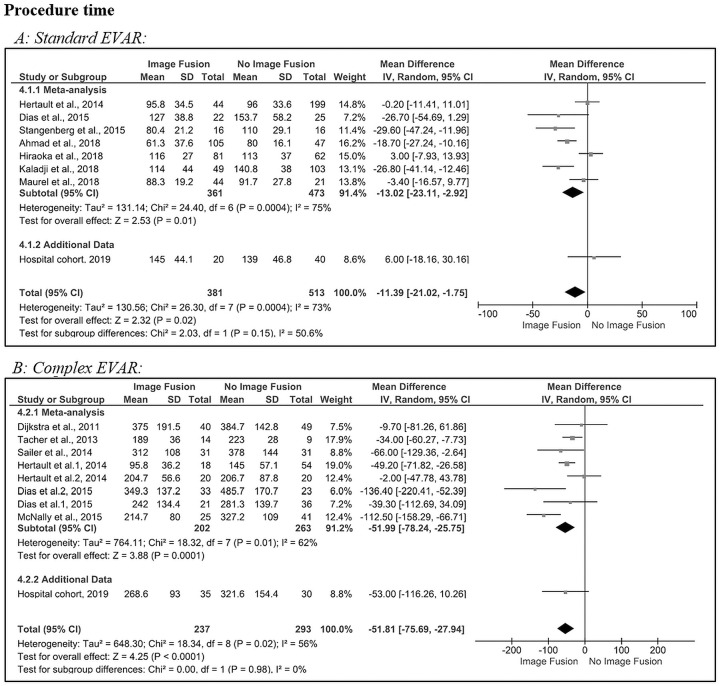
Forest plots of procedure time (minutes) for (A) standard and (B) complex endovascular aneurysm repair (subgroups were analyzed for Dias et al^6^ and Hertault et al^4^). CI, confidence interval; IV, inverse variance; SD, standard deviation.

Figure 5A shows the pooled results for the procedure time used in standard EVAR procedures. The estimated pooled MD revealed a statistically significant decrease in procedure time after image fusion compared to no image fusion (–11 minutes, 95% CI −21.0 to −1.8, p=0.02). There was a significant heterogeneity (Q=26.3, p<0.001; *I*^2^=73%) in the 7 included studies^4,6,17–21^ and the hospital cohort. The standard EVAR hospital cohort MD (+6 minutes, 95% CI −18.2 to 30.2) was comparable to Hiraoka et al^18^ (+3 minutes, 95% CI −7.9 to 13.9).

In complex EVAR (Figure 5B), statistical heterogeneity was significant, and the between-study variability was considered moderate (Q=18.3, p=0.02; *I*^2^=56%) in the 6 included studies^4,6–10^ and the hospital cohort. The estimated pooled MD revealed a significant difference in procedure time after image fusion compared with no image fusion (–52 minutes, 95% CI −75.7 to −27.9, p<0.001). The complex EVAR hospital cohort MD (–53 minutes, 95% ‒116.3 to 10.3, p=0.11) was comparable to Hertault et al^4^ (–49.2 minutes, 95% ‒71.8 to −26.6, p=0.03). The stent-graft configurations consisted of 234 fenestrations and/or branches (average 3.1 per case). In total there were 46 fenestrated cases, 21 branched cases, and 6 fenestrated/branched cases. Also, there were 20 stent-graft configurations with scallops and 4 with proximal cuffs (without iliac limbs).

## Discussion

Image fusion is becoming a more widely utilized imaging tool during aortic endovascular procedures. However, to date little has been published about the advantages of image fusion during standard EVAR in comparison to complex EVAR. This literature review provided evidence that image fusion reduces the amount of contrast volume and procedure time for both standard and complex EVAR procedures. The contrast volume reduction is larger for complex EVAR (–79 mL, p<0.01) than in standard EVAR (–29 mL, p<0.01), which seems plausible since visualization of the arterial ostia with iodinated contrast has been replaced by on screen navigation. The meta-analysis showed a significant decrease in fluoroscopy time for complex EVAR (–14 minutes, p=0.009), while fluoroscopy time was not reduced in standard EVAR. Most studies providing data on radiation exposure suggested a dose reduction, which was reflected in the current meta-analysis, though it did not reach statistical significance.

Regarding procedure times, the pooled results of all studies showed a larger reduction in the complex EVAR group (–52 minutes, p<0.001) compared with standard EVAR (–11 minutes, p=0.02). An explanation for this is unknown; however, from our own experience, cannulation time of visceral arteries can be shortened with the use of image fusion.

The hospital cohort showed the most comparability with the results of Hiraoka et al^18^ for standard EVAR, with minor differences between fusion and no fusion. For complex EVAR, a large procedure time reduction was observed, although it was not significant. This is in line with the pooled data.

### Limitations of the Outcome Parameters

When comparing the procedure metrics of image fusion with the control groups, a risk of bias is introduced. First, contrast volume reduction during EVAR relies heavily on the digital subtraction angiography (DSA) protocol choice. In this review, there were protocols with varying contrast volumes and flows between 30 mL at 15 mL/s and 7 mL at 30 mL/s. Not all papers noted their DSA protocol and not all articles noted the concentration of iodine in mg/mL of the administered contrast agent. To adequately compare fusion with no fusion, the contrast protocol should not be changed after implementing image fusion. Many studies did not give data on the contrast protocol before and after implementation of image fusion.

Second, fluoroscopy time is measured from the start of the first X-ray pulse until the end of the last pulse. The difficulty with this metric is that it is operator dependent, whereas with contrast volume a protocol can be followed. During complex EVAR, fluoroscopy is needed to visualize the catheter tip and region of interest while cannulating the visceral arteries. The only hypothesized reduction in fluoroscopy time due to image fusion is when the C-arm and table are moved to a specific region of interest, when the 3D roadmap is used without fluoroscopy, and when pre-planned C-arm angles are utilized.

Third, proper radiation dose reduction needs a multifactorial approach. The largest contributors to high radiation doses in EVAR are the amount of DSA performed per procedure and a high patient body mass index. Also, C-arm X-ray settings, such as the frames per second, collimation (smaller field of view), and fluoroscopy protocol,^12,22–24^ can be major factors in radiation dose reduction. Not all articles noted their radiation dose protocols in terms of the aforementioned parameters. Dias et al^6^ describes a “combined approach” in which image fusion is implemented together with radiation dose reduction strategies.

Additionally, the registration type of image fusion can be performed by matching bony landmarks (2D-3D registration) or by matching renal and aneurysmal calcifications (3D-3D registration). Dijkstra et al^7^ reported the exposure to radiation of 40 patients during f/bEVAR performed using 3D-3D registration; the 3D-3D registration contributed a median radiation dose of 290 mGy (interquartile range 270, 310), which is 7-fold higher compared with our institution (median 44 mGy, interquartile range 33, 55). Stangenberg et al^21^ preferred 3D-3D registration because of its suggested higher accuracy even though it required more radiation. In our center, 2D-3D registration is preferred for standard EVAR given the lower registration dose compared to the total procedure radiation dose. The 3D-3D registration is used mostly for complex EVAR since image fusion is more useful during cannulation of the visceral arteries. The advantage of higher accuracy without manual correction, especially in lateral views, outweighs the disadvantage of a higher registration dose, which is comparable to a single DSA. Another reason is that the total procedure radiation dose for complex EVAR is on average 2 to 3 times higher compared to standard EVAR based on the data in this meta-analysis. The higher registration dose introduced by 3D-3D registration is a relatively small increase in total procedure radiation dose during complex EVAR.^12^

Fourth, the definition of procedure time is subject to debate and bias. Only the article of McNally et al^8^ described it as time from skin incision/puncture to bandage application. Moreover, most of the included studies mentioned explicitly that all patients were operated by experienced operators to minimize the effect of the learning curve. However, especially for complex EVAR, a learning curve is still present even for experienced operators given the relatively short period of time that fenestrated/branched stent-grafts have been available. In the study of Hertault et al,^4^ the procedures were performed by vascular surgeons in training as well as by experienced physicians, which may explain the absent decrease in procedure time in the fEVAR group, which can be seen in Table 2.

Additionally, procedure time can be influenced by many factors, such as operator experience, but also by the complexity of the stent-graft and the procedure and other perioperative variables. These important metrics were mentioned only by McNally et al^8^ and included blood loss, length of stay, and renal function among others. In their study, the use of image fusion appeared to favorably impact these perioperative outcomes.

### Study Limitations

The majority of studies^6-8,10,17,18,21^ in this analysis had a retrospective design, and the matching process to obtain the control group was not consistent in all studies, which is likely to have influenced the outcomes. The selection of patients might have resulted in a different outcome if all procedures had been subdivided based on complexity/number of fenestrations/branches. This selection bias also applies to our own hospital data since the control group was retrospective.

It is worth noting that all operators were aware of the possible outcome measures such as contrast volumes and radiation doses, which means they might have paid close attention to these outcomes and thus lowered the amount of contrast and/or radiation dose registered. Furthermore, the current analysis is limited by the influence of operator experience on the learning curve, the relatively small patient numbers, the moderate heterogeneity among the study groups, and missing data.

Moreover, reporting standards were not uniform. Radiation dose parameters were reported as cumulative AK or DAP. AK is an approximation of the total radiation dose to the patient skin, whereas DAP is the total X-ray energy leaving the X-ray tube. From the meta-analysis, the majority of studies reported DAP and so that measure was used in the pooled data analysis.

Additionally, there are some inevitable differences between the presented studies, notably in hardware. The various hybrid operating room vendors might have influenced the radiation dose since this parameter is generated by the X-ray source. Furthermore, the use of a mobile or fixed C-arm can influence the radiation dose, as described by de Ruijter et al.^12^ However, since most studies in the meta-analysis focused on mean differences between fusion and control groups under mostly identical circumstances, bias due to different hardware or mobile/fixed C-arms can be considered minimal.

A separate potential bias is the volume of patients treated per center and operator. Most studies in the meta-analysis did not report their annual patient numbers for standard and complex EVAR, which could influence fluoroscopy and procedure times according to operator experience.

This meta-analysis contains only a modest sample of 11 studies with individual participant data, and methodological deficiencies, such as subgroup analysis and publication bias, could not be properly explored due to a lack of data. However, these results are sufficient to show that extensive research on image fusion during complex EVAR is needed, especially in terms of clinical success, decreasing radiation exposure, and long-term outcomes of the procedure. The use of iodinated contrast is currently indispensable during these procedures, especially in complex EVAR. Alternatives should be investigated, especially for patients who suffer from nephropathy. For instance, Dias et al^6^ selectively injected carbon dioxide in one of the renal arteries. Taking advantage of the slower reflux of this gas into the aorta, this lowered the DSA frame rate to 2 frames per second.

Moreover, advances can be made in image fusion registration workflow. Currently with 2D-3D or 3D-3D registration, manual interaction is needed to link the preoperative imaging with fluoroscopy. This can be tedious and time-consuming due to lack of experience.^12^ An automated registration algorithm could exceed human capabilities by integrating a self-learning principle via machine learning.^25^ Another improvement would be the use of algorithms to correct for vessel displacement (mostly iliac artery) after insertion of stiff guidewires, sheaths, and stent delivery devices to ensure a one to one 3D roadmap that continuously corrects for anatomic vessel changes.^26–33^ With algorithms like these, the user can rely more on image fusion during the procedure. The need for large contrast volumes in order to navigate might be completely eliminated, and only small volumes of nephrotoxic contrast would be needed for image fusion validation.

## Conclusion

The results of this meta-analysis confirm that image fusion significantly reduces contrast volume, fluoroscopy time, and procedure time in complex EVAR but only contrast volume and procedure time for standard EVAR. Though a reduction was suggested, the radiation dose was not significantly impacted by the use of fusion imaging in either standard or complex EVAR.

## Appendix A

### Database-Specific Search Queries

#### Medline

((“Endovascular Procedures”[Mesh] OR endo-vascular[tiab]) AND (((aortic[tiab] OR “Aneurysm”[Mesh] OR aneurysm*[tiab]) AND (repair*[tiab] OR morphol*[tiab] OR anatom*[tiab])) OR evar[tiab] OR evas[tiab] OR bevar[tiab] OR chevar[tiab] OR fevar[tiab] OR pevar[tiab] OR tevar[tiab])) AND (“Imaging, Three-Dimensional”[Mesh:noexp] OR “Radiography, Interventional”[Mesh] OR “Surgery, Computer-Assisted”[Mesh] OR ((“3 d”[tiab] OR 3d[tiab] OR 3dus[tiab] OR “3 dus”[tiab] OR 3dimension*[tiab] OR ((three[tiab] OR 3[tiab]) AND dimension*[tiab])) AND fusion[tiab]) OR (image[tiab] OR images[tiab] OR imaging[tiab] AND fusion[tiab]) OR hybrid fusion[tiab])

#### Embase

(‘endovascular surgery’/exp OR endovascular:ab,ti,kw) AND ((aortic:ab,ti,kw OR ‘aneurysm’/exp OR aneurysm*:ab,ti,kw) AND (repair*:ab,ti,kw OR morphol*:ab,ti,kw OR anatom*:ab,ti,kw) OR evar:ab,ti,kw OR evas:ab,ti,kw OR bevar:ab,ti,kw OR chevar:ab,ti,kw OR fevar:ab,ti,kw OR pevar:ab,ti,kw OR tevar:ab,ti,kw) AND (‘three dimensional imaging’/de OR ‘interventional radiology’/exp OR ‘computer assisted surgery’/exp OR ((‘3 d’:ab,ti,kw OR 3d:ab,ti,kw OR 3dus:ab,ti,kw OR ‘3 dus’:ab,ti,kw OR 3dimension*:ab,ti,kw OR ((three:ab,ti,kw OR 3:ab,ti,kw) AND dimension*:ab,ti,kw)) AND fusion:ab,ti,kw) OR ((image:ab,ti,kw OR images:ab,ti,kw OR imaging:ab,ti,kw) AND fusion:ab,ti,kw) OR ‘hybrid fusion’:ab,ti,kw)

#### Cochrane

(((aortic:ab,ti,kw or aneurysm*:ab,ti,kw) and (repair*:ab,ti,kw or morphol*:ab,ti,kw or anatom*:ab,ti,kw)) or evar:ab,ti,kw or evas:ab,ti,kw or bevar:ab,ti,kw or chevar:ab,ti,kw or fevar:ab,ti,kw or pevar:ab,ti,kw or tevar:ab,ti,kw) and (endovascular:ab,ti,kw) and (“interventional radiolog*”:ab,ti,kw OR “computer assisted surg*”:ab,ti,kw OR ((“3 d”:ab,ti,kw OR 3d:ab,ti,kw OR 3dus:ab,ti,kw OR “3 dus”:ab,ti,kw OR 3dimension*:ab,ti,kw OR ((three:ab,ti,kw OR 3:ab,ti,kw) AND dimension*:ab,ti,kw)) AND fusion:ab,ti,kw) OR (image:ab,ti,kw OR images:ab,ti,kw OR imaging:ab,ti,kw AND fusion:ab,ti,kw) OR “hybrid fusion”:ab,ti,kw)

## Appendix B

### Details of Preoperative Imaging Acquisition and Postprocessing, Registration, and Image Fusion for the Hospital Cohort

#### Preoperative Imaging

Prior to all procedures, a CTA scan of each patient was acquired at 120 kV and 130 mA·s with 512×512×~1100 voxels (voxel size 1×1×0.96 mm) and 100 mL of 300-mg I/mL contrast (Xenetix 300; Guerbet, Aulnay-sous-Bois, France). In the fusion and no fusion cohorts, the iodinated contrast protocols were similar in terms of volume and flow during administration. Vessel Navigator guidance (Philips Healthcare) was utilized in the image fusion cohort. The CTA images were loaded into a vascular workstation (Philips Interventional Tools r9.0) for automated vessel segmentation. The following arteries were manually selected for complex EVAR: the aorta, celiac artery (CA), superior mesenteric artery (SMA), right and left renal artery (RRA/LRA), possible accessory renal arteries, right and left common iliac arteries (RCIA/LCIA), and right and left internal iliac arteries (RIIA/LIIA). The ostia of the CA, SMA, RRA, LRA, RIIA, and LIIA were identified and manually marked with circular navigation markers placed orthogonal to the vessel lumen and corrected in the axial, sagittal, and coronal planes. The CA and SMA were excluded in standard EVAR. When heavy calcification at the vessel ostia was present, extra attention was paid to whether the navigation marker was placed in the true lumen by scrolling through the axial CT plane. The C-arm angles were determined by viewing the aortic branch vessels at a perpendicular angle to their origin, and navigation markers were placed by moving the virtual C-arm. The selected angles were saved for immediate recall during the procedure.

#### Registration and Live Guidance

For standard EVAR, two 2D fluoroscopy images were acquired with an angular difference ≥30° in the right and left anterior oblique planes. The 90° right anterior oblique and the 0° anteroposterior views were typically used in the hybrid operating room with the ceiling-mounted C-arm system (Azurion Flexmove 7 C20; Philips Healthcare). After the 2 fluoroscopy acquisitions, the registration was performed on the workstation while the patient was iodinated and draped. The 2D-3D registration was performed with alignment of the vertebral column and proximal pelvic rings, with the field of view centered on L1/L2 with registration emphasis on the vertebral column.

For complex EVAR, 3D-3D registration was performed since this relies more on millimeter image fusion accuracy during cannulation of the visceral arteries. With 3D-3D registration, a cone-beam computed tomography is performed intraoperatively. The C-arm follows a 180° circular trajectory that results in a 3D scan that is registered to the preoperative CTA by matching at least 5 renal and aneurysmal calcifications. Calcifications more distal in the iliac trajectories were excluded due to possible mismatch.

For all cases, registration was performed after patient draping and before the insertion of stiff sheaths and guidewires. After this, the preoperative CTA was fused with the fluoroscopic image stream and functioned as live guidance. After the introduction of the sheath, guidewires, diagnostic catheter, and stent-graft delivery system, a DSA was performed using an automatic iodine pump (20 mL at 15 mL/s of 300-mg I/mL UltraVist; Bayer HealthCare AG, Berlin, Germany) to visualize the ostium of the renal arteries and proximal sealing zone. When necessary, a manual correction was performed by translating the image fusion overlay to match the true ostium of the renal arteries on the DSA. From this moment, the image fusion overlay was kept fixed, and no manual correction was performed except in extreme lateral projections.

To visualize the internal iliac arteries, 10 mL of diluted contrast or 5 mL of undiluted contrast was administered, depending on the operator’s choice. At the end of each procedure, the contrast volume and procedure time (incision to wound closure) was recorded, and an automated dose report was created that included the patient radiation dose (cumulative AK), DAP, and fluoroscopy time. In the complex EVAR cohort, only procedure time was recorded in the control group and data on contrast volume, fluoroscopy/procedure times, and radiation dose were unavailable.
